# Exploring People’s Knowledge of Genetics and Attitude towards Genetic Testing: A Cross-Sectional Study in a Population with a High Prevalence of Consanguinity

**DOI:** 10.3390/healthcare10112227

**Published:** 2022-11-07

**Authors:** Amal Alotaibi, Njoud Khaled Alkhaldi, Areej Mustafa AlNassir, Leenah Ayman AlAyoubi, Nada Abdulrahman AlMalki, Rahaf Abdullah Almughyiri, Reem Hussain AlDosary, Mary Anne Wong Cordero

**Affiliations:** College of Medicine, Princess Nourah bint Abdulrahman University, Riyadh 11671, Saudi Arabia

**Keywords:** knowledge of genetics, genetic testing, consanguineous marriage, carrier screening

## Abstract

This study investigated people’s knowledge of genetics, attitudes toward genetic testing, and views on consanguinity. This cross-sectional study utilized a validated questionnaire modified from published studies to collect data on people’s knowledge of genetics and attitudes about genetic testing among 1008 respondents from various Saudi Arabian regions. Using SPSS software version 26, data were analyzed using a *t*-test, ANOVA, and multivariate analysis. *p*-values of <0.05 were considered statistically significant. About 59.9% of the participants had sufficient knowledge of genetics, and around 50% had a favorable attitude toward genetic testing. Knowledge of genetics is significantly correlated (*p* ≤ 0.001) with a positive attitude toward genetic testing. Gender, age, level of education, marital status, family income, and family history were significantly correlated with respondents’ understanding of genetics. Gender, family income, residence, and family history were associated with attitudes toward genetic testing at a 0.05 level of significance. There is a need to strengthen peoples’ knowledge of genetics and attitudes toward genetic testing through diverse educational programs and healthcare strategies. Impetus on how to disseminate genetic information on consanguinity and transmission of diseases should be prioritized in regions where consanguineous marriages are high.

## 1. Introduction

The importance and impact of genetics in medicine have grown with the discovery of genetic susceptibility to common conditions such as cancer, cardiovascular disease, and even psychiatric illness. The discoveries of rare genetic illnesses and disorders have only added to this; hence, genetics should be taught in every medical school [1]. Genetic literacy is a type of health literacy that incorporates knowledge of basic human genetic ideas and medical genetics. As a result, it is necessary to provide adequate care to individuals with genetic abnormalities [2]. Healthcare providers are now faced with novel genetic techniques that necessitate genetically informed judgments impacted by patients’ genetic knowledge and attitudes toward genetic testing [3,4].

Completing the Human Genome Project (HGP) paved the ground for a quick acknowledgment of genetics’ importance in medicine. Advances in genetics and genomics, particularly human genome sequencing, have prepared the way for a paradigm change in illness prevention, diagnosis, and treatment based on a person’s genetic profile [5]. The accomplishment of the HGP has revolutionized clinical approaches to diagnosis [6]. Genomic testing is now being used for tumor molecular characterization, preconception carrier screening, prenatal and postnatal atypical condition detection, and infectious illness diagnosis [7]. We may soon be able to use gene-editing technology to predict illness risk accurately, customize existing treatments based on hereditary and non-genetic factors, and cure or even eradicate some diseases [8]. An average of ten novel genetic testing items is generated daily, demonstrating that genomic testing is becoming more popular in clinical practice [9]. Due to technological breakthroughs, lower testing prices, and increased public knowledge, genetic services are in high demand in clinical and direct-to-consumer (DTC) settings [10]. Genetic testing has been significantly used in medical practice to deliver individualized care. The new vision of genetic research is to advance genetic testing so that it is used to deliver individualized care based on a patient’s genetic makeup and to predict possible genetic disorders within the family [11,12].

It should be understood that genetic testing for health risks in a particular population has benefits and drawbacks. It has advantages for disease diagnosis, family planning, medical research, and other facets of healthcare. However, there are limitations to screening, and risk detection is one of them. Another is the complexity of deciding what course of action to take in light of that risk [13]. Despite being a valuable tool for preventing the spread of many inheritable diseases, expanded carrier screening (ECS) is not widely utilized for pregnancy risk assessment. This is most likely a result of the early application’s inconsistent use of good practice suggestions [14]. In addition, there are specific recurrent technological problems, such as inaccurate interpretation of genetic variations [15] and insufficient education on the fundamental genetics among patients and the medical community. Clinicians and health care policymakers must therefore consider the risks and benefits associated with test use as well as how accurately a test identifies a patient’s clinical state (clinical validity) when assessing the proper use of new genetic tests (clinical utility) [16]. The accuracy and potential for improving health outcomes of genetic tests already in use vary. These test features may be impacted by testing technology and the clinical environment in which the test is utilized [16,17].

In several Middle Eastern countries, consanguineous marriages are common; genetic disorders are prevalent and have more severe effects among the Arab populations [18,19,20]. This condition may bring up moral, social, and legal concerns specific to a particular group of people or community. Previous research has shown that various characteristics, including age, gender, and educational attainment, influence public opinions toward genetics and genetic testing [20,21]. In Saudi Arabia, genetic diseases are widespread in the general population. This high incidence is linked to consanguineous marriages and large families, advanced maternal age, and a lack of healthcare efforts to prevent such disorders [22,23,24,25]. The country introduced the first National Premarital Screening Program (NPMS) in 2014 to reduce the risk of inherited disorders similar to hereditary hemoglobin abnormalities. This program helps limit the danger of genetic diseases passing down through the generations [3,26,27,28,29]. Despite being required for the application of a marriage certificate, all couples with marriage proposals have the freedom not to complete their marriage, whatever the results of NPMS [26,27]. Saudi Arabia now offers clinical diagnostic, therapeutic, and preventative programs such as neonatal, premarital, and preimplantation genetic diagnosis [27]. Added to the different genetic services within the Kingdom is the Saudi Human Genome Program (SHGP), the largest genome initiative in the Middle East, which aims to reduce and prevent genetic diseases [25]. 

There needs to be more research on the general public’s knowledge of genetics and attitude toward genetics testing among the Saudi population. A fundamental understanding of genetics is necessary to comprehend genetic testing and its potential benefits for people. Comprehending people’s attitudes about genetic testing can help address the challenges, misunderstandings, and information gaps in this subject. This study investigated people’s knowledge of genetics, their attitudes regarding genetic testing, and the factors influencing their decisions. The findings of this study could be used to improve educational programs and healthcare promotion strategies aimed at increasing public knowledge and awareness of genetics and genetic testing. 

## 2. Materials and Methods

This study is a cross-sectional, paper-and-pencil-based questionnaire survey that explored the extent of people’s knowledge about genetics and attitudes toward genetic testing. A random sampling technique was used, and the sample size was calculated using G-power. The expected prevalence of adequate knowledge ranged between 42.2% and 50%, with a power of 80% (β=0.2) and a confidence level of 95% (α=0.05). Calculation using the G power program showed a minimal sample size of 998, but to compensate for missing and incomplete data in the questionnaire, the recruitment of 1015 respondents was organized. Participants were male and female aged eighteen and above, irrespective of their education and socioeconomic status. Data were collected for almost three months, from 1 November 2019 to 23 January 2020, from various places, such as shopping malls, schools, universities, hospitals, libraries, and other public places in the regions of the Kingdom of Saudi Arabia (KSA). This was conducted by approaching possible random respondents. Before the participants were asked to complete the survey, brief information about the aim and objectives of the research was presented. The voluntary nature of the survey, participants’ absolute right to refuse to answer the survey questionnaire at any time, and the confidentiality of the data were explained. The survey took an average of 8 to 13 min to complete for each participant. After finishing the survey, the participants received no reward.

### 2.1. Research Instrument

People’s knowledge of genetics and attitudes toward genetic testing was determined using questionnaires adapted and modified from previously published studies [29,30,31]. The questionnaire was translated into Arabic following the backward translation method to indicate semantic equivalence and enhance the validity of the questionnaire. Two independent bilingual professionals translated the questionnaire into Arabic, after which the researchers evaluated the quality of the translation. Discrepancies in translation were noted and resolved by discussing them with the translators. Backward translation was performed by two independent translators who had not seen the original questionnaire. The Arabic version of the survey questionnaire was pilot tested on 30 persons of various demographic profiles. Feedback from the pilot study was utilized as the bases for necessary revisions, such as rewording two questions and improving the clarity of instruction. The data from the pilot test were not included in the final results. 

The questionnaire was divided into three sections: The respondent’s profile, knowledge of genetics, and attitude toward genetic testing. Respondent’s profiles included age, gender, level of education, marital status, and family income. Participants were also requested to indicate from what region in KSA they live and whether or not they were born from a consanguineous marriage.

General knowledge of Genetics was assessed using fifteen (15) structured true or false questions, including basic genetic concepts, inheritance of diseases, the interaction between genes and environment, and genetic variation. Survey questions to estimate the current level of genetic knowledge among Saudis were adapted from previous studies [29,30]. The first five items in the questionnaire assess basic genetic knowledge relevant to genes and heredity, while items 6 to 11 evaluate basic concepts of the inheritance of genetic diseases. Items 12 to 14 included statements that gauged peoples’ understanding of genetic variation and the influence of other factors, such as environment, lifestyle, and ethnicity in the expression of genetic disorders. Due to the high frequency of consanguinity in KSA, one item (number 15) on consanguineous marriage concerning genetic disease was included. This inclusion was deemed essential to determine whether the participants knew the genetic risks accompanying consanguineous marriages. 

In assessing attitudes toward genetic testing, participants were required to complete 12 questions related to their attitude toward genetic testing. Items 1 to 7 assessed peoples’ attitudes toward Predictive Genetic Testing (PGT). This test intends to determine a person’s genetic history and condition and whether they are at risk for a genetic disorder. It investigates genetic alterations or mutations related to diseases that occur before a person exhibits any symptoms of an illness [1]. Items 1,2, and 4 are positive statements, while 3,5 and, 6 are negative statements. Items 8 to 12 focused on the public’s attitude towards Preconception Carrier Testing (PCT). This test can identify whether a person carries the gene for a particular genetic disorder and determines the likelihood that he or she will convey a genetic disorder to his or her progeny [1]. Questions to gauge participants’ opinions about genetic testing were adapted to suit the Saudi community and derived from a previously published survey. On a scale of 1 to 4, answers to the questions ranged from strongly disagree to strongly agree. Additionally, respondents were questioned about their willingness to submit to premarital genetic testing even if it is not required. One question about the cancelation of marriage due to a genetic test was added because arranged marriages are practiced in KSA. The total knowledge score ranged from 0 to 15. Correct responses to knowledge questions were assigned a score of 1 and 0 for wrong answers. Respondents who scored nine and above (60% and above) were considered to have adequate genetics knowledge. The total attitude score ranged from 0 to 12. Positive answers were scored as 1, and negative answers were scored as 0. Respondents with a score of ≥7.2 (60%) or above were considered to have a positive attitude toward genetic testing. 

### 2.2. Data Analyses

Only completed survey questionnaires (1008) were included for data processing and analysis. Descriptive statistics in terms of means, standard deviations, median and interquartile ranges were used to describe the criteria of the studied sample. Analysis of quantitative data by ANOVA and association of qualitative variables by chi-square test was conducted. A multivariate analysis was used to determine the relationship between family history, whether children were born consanguineously or not, and other characteristics as predictors of genetic knowledge and attitudes toward genetic testing. *p*-values of less than 0.05 were considered statistically significant. All statistical analysis was conducted using Statistical Package for the Social Sciences (SPSS) software version 26.

## 3. Results

### 3.1. Sociodemographic Characteristics 

A total of 1008 completed questionnaires were collected from various regions in Saudi Arabia. A summary of the sociodemographic characteristics of respondents is presented in Table 1. Most of the study participants were below 30 years (65%), single (61.9%), college students or with a college degree (63.6%), and with good socioeconomic income (87.4%). Slightly more than half of the respondents were female (52.9%), and most respondents were from Riyadh (77.1%), the capital city of Saudi Arabia. The consanguineous marriages among the respondents’ parents were almost half (46.7%).

### 3.2. Knowledge of Genetics

People’s general knowledge of genetics is summarized in Table 2. Overall, study participants scored 8.98 out of 15 (59.9%) as assessed by the 15 item genetics knowledge questionnaire. Worth noting are genetic concepts related to genes (items 3 and 4) and inheritance of disease (items 9 and 10), which most respondents were unaware of and thus scored low. Our findings also revealed that most respondents (80.2%) knew that consanguineous marriages increase the possibility of having children with genetic diseases. 

### 3.3. Attitudes toward Genetic Testing

Table 3 presents respondents’ attitudes toward genetic testing. Study participants generally scored more than 7.2 out of 12 or 60% and above, indicating a positive attitude towards genetic testing. For attitudes toward Predictive Genetic Testing (PGT), the majority expressed curiosity about their genetic predisposition to diseases. They were willing to take a genetic profiling test to determine whether they risk developing inherited diseases. For Preconception Carrier Testing (PCT), many respondents believed couples planning pregnancy should undergo carrier tests. They expressed those newborn children should be genetically tested for possible diseases they may develop when they become adults. However, some respondents were worried that the results of a genetic test may fall into the wrong hands (58.3%) and may lead to higher anxiety among women who want to become pregnant (63.2%). 

### 3.4. Premarital Genetic Testing

**Figure 1:**
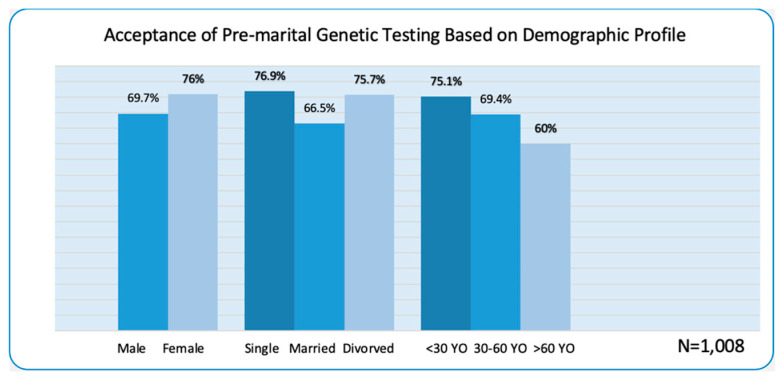
Acceptance to Premarital Genetic Testing Based on Demographic Profile.

As shown in Figure 1, most (60% to 76.9%) study participants have a positive attitude and acceptance toward premarital genetic testing. More females (76%) expressed willingness to undergo the testing even if it is not mandatory. The graph also shows that single respondents have the highest acceptance (76.90%) of premarital genetic testing. The figure likewise indicates a high acceptance of premarital genetic testing among people below 30. 

### 3.5. Association between Knowledge of Genetics and Attitudes to Genetic Testing with Socio-Demographic Profile

Results showed that participants’ knowledge of genetics is significantly correlated (*p* ≤ 0.001) with a positive attitude towards genetic testing. Those with a higher knowledge level in genetics showed a more positive attitude toward genetic testing. Table 4 displays the relationship between the participants’ sociodemographic characteristics and their overall genetics knowledge. The findings show that, at a 0.05 level of significance, the respondents’ knowledge of genetics was significantly associated with gender, age, education level, marital status, family income, and family history. Female participants were significantly more knowledgeable in genetics when compared with male participants. Those below 30 years old, in college and post-graduate, and earning enough for a family, have a higher level of knowledge in genetics. Single, married, and divorced respondents showed an adequate knowledge of genetics.

The relationship between the examined sociodemographic and attitudes toward genetic testing is also shown in Table 4. Results show a significant influence of gender, family income, residence, and family history, whether born from consanguineous or non-consanguineous marriage, with the respondents’ attitude towards genetic testing at <0.05 significant level. Females and those having enough income showed positive attitudes toward genetics testing.

The multivariate analysis in Table 5 shows that gender and the level of education are predictors of both knowledges of genetics and attitude toward genetic testing. Additionally, respondents born from consanguineous marriages may predict attitudes to genetic testing but not knowledge of genetics.

## 4. Discussion 

The key outcomes in this study were the public’s knowledge of genetics and attitude toward genetic testing. Our investigation revealed that 59.9% of the respondents understood genetics adequately. This result is higher when compared with the Jordanian population, in which 43.4% of the 5000-population surveyed were knowledgeable in genetics [32]. However, this number is lower than that of a survey involving 920 students at a Saudi Arabian university, which indicated that more than 63% of the participants had adequate knowledge of genetics [3]. However, it is essential to mention that the study’s participants were university students, and it is safe to assume that they learned the fundamentals of genetics as part of their biology or genetics classes. 

This study demonstrated that knowledge scores on genetics were influenced by gender, marital status, and the level of education. It has been found that women are more knowledgeable about genetics than men, which is strengthened by the participant’s educational background [32,33]. Women, especially mothers, regularly worry about their health, and how it may affect their lives and their children’s lives, which is the most likely explanation for this finding. Additionally, those with advanced degrees (college and post-graduate) had a much higher overall genetics knowledge score, consistent with findings from earlier research [12,32,34,35]. Results of the current study likewise revealed that those with a sufficient or more significant family income have a higher genetics knowledge score. This result parallels findings from a prior study, which found that higher income and a higher education were correlated with better knowledge of genetics [32,36]. Our research also revealed that, compared to other age groups, young, single, and unmarried people were better aware of genetics. This might be accounted for by the fact that most responders in this age group are post-graduate or college students. 

Further analysis of the sociodemographic characteristics through multiple regression revealed that gender and the level of education are predictors for both knowledge in genetics (*p* = 0.000) and attitude towards genetic testing (*p* = 0.026). Our findings suggest that a highly educated public will conform to genetic testing more. The difference in the genetic knowledge and attitudes toward genetic testing is worth noting when analyzed according to a family history. Results showed a significant difference (*p* = 0.005) in attitudes toward genetic testing between respondents who were born from consanguineous marriages and non-consanguineous marriages. Those born from consanguineous marriages have positive attitudes toward genetic testing but need to be more knowledgeable about genetics. Our results also showed that most respondents (80.2%) knew that consanguineous marriages increase the likelihood of producing children with a genetic disorder. The younger Saudi generation, with a high level of education and a good knowledge of genetic disorders, has diminishing support for consanguineous marriage. Despite this finding, the prevalence of consanguineous marriage is still high in KSA. The possible reason is the strong influence of customs and traditions that still prevail and support consanguineous marriages. It was recognized that family tradition is essential in the pervasiveness of consanguinity and genetic disorders in Saudi Arabia [37]. 

Consanguinity in Saudi Arabia is heavily influenced by beliefs and tradition, which presents a significant challenge to the healthcare and educational sectors and the nation as a whole, as it undergoes enormous cultural changes as part of Vision 2030. Some of these reforms include removing public segregation of the sexes, lifting the ban on women driving, and religious police losing their authority [38] [Ranaa Madani, 2022]. The Ministry of Health (MOH) is part of the National Transformation Program’s vision. One of its main objectives is to improve the quality of healthcare services using prevention and therapeutic approaches to control diseases. The Kingdom will experience a significant change in the upcoming years as it modernizes its healthcare system by implementing several initiatives to inform the populace and raise awareness of critical health issues, for example the burden that genetic abnormalities place on society’s health. Although there will be challenges and more time to educate people about the effects of consanguineous marriages, the optimistic attitudes toward genetic testing, found in the current study and previous studies, may render cause for confidence.

Nearly half of the study participants had a positive attitude toward predictive and preconception carrier testing. Respondents with an adequate understanding of genetics had a favorable attitude toward genetic testing. Based on Rosenstock’s Health Belief Model, people’s health-related beliefs and behaviors depend partly on their knowledge level, which explains how education may influence people’s attitudes [39]. The model tries to describe conditions under which a person will engage in specific health behaviors, such as preventive screening or getting treatment for a medical condition [39]. In the current study context, respondents with adequate knowledge of genetics manifest positive attitudes toward genetic testing. Our results also reveal gender (*p* = 0.001), family income (*p* = 0.001), and place of residence (*p* = 0.047) to be associated with attitudes towards genetic testing. Those living in urban areas have a more positive attitude towards genetic testing than those in rural regions. This may be due to some rural regions’ underutilization of genetic testing and counseling services. The primary healthcare practitioners’ referral and utilization of these services could be improved by genetics education [40]. It must be considered, however, that about 70.1 % of the sample in our study is from an urban location and only 22.9% from rural regions. This underrepresentation of respondents from rural areas may have some influence on people’s actual attitudes toward genetic testing.

In other studies, the public’s perception of genetic testing for assessing illness risk is largely favorable [32,41,42,43]. In addition, a study in Riyadh on public perception and awareness of genetic testing found that 87% of the 333 respondents expressed they would consider genetic testing before marriage and would not consider having a child if genetic screening revealed a 100% chance the child would be born with a genetic disorder [44]. A study in Belgium reported that the majority of the 1182 respondents had a moderate interest in PGT and were willing to be tested solely for treatable or preventable diseases. They are willing to undergo genetic tests for reproductive purposes, such as preconception recessive disorder screening and prenatal genetic testing [31]. In a recent study on public attitudes toward genetic testing in Georgia, most respondents were interested in PGT and preferred to be tested only for treatable or preventable disorders. They also expressed interest in having their newborn children tested for late-onset disorders and having preconception carrier screening [45]. These findings are similar to those of our study. Nearly half of the participants had a favorable attitude towards predictive genetic testing. They were willing to take a genetic profiling test to determine their risk of acquiring disorders.

Furthermore, many respondents agreed that PCT is reasonable and that couples contemplating pregnancy should take a carrier test. Our findings reveal that gender and family income are associated with a favorable attitude towards genetic testing. Female participants with a higher income had a more optimistic view of carrier testing, predictive, and preconception. In a study of senior college students at one Saudi Arabian university, most students had favorable sentiments toward genetic testing [3]. Likewise, our study included people aged 18 to over 60, and most of them (63.6%) were college students.

On the other hand, some apprehensions and negative attitudes concerning genetic testing, voiced by nearly half of the study participants, must be addressed. To cite, many respondents were concerned that genetic testing might result in the annulment of their marriage. They also expressed concern that carrier testing could negatively portray persons with inherited disorders and that disabled people would be less accepted in society. These unfavorable attitudes and misconceptions about genetics and genetic testing can be clarified and addressed by educating and enhancing peoples’ knowledge of genetics and genetic testing. One research study among Jordanian women found that they are open to the countrywide deployment of non-invasive prenatal genetic screening, with the caveat that it should be accompanied by health education to improve the population’s genetic literacy [46]. 

Public cognizance of genetic risks associated with consanguinity is indispensable. Various research has shown that consanguinity affects a person’s health over many generations and is a serious public health concern [20,47,48]. This study reports a high prevalence of consanguinity in nearly half (46.7%) of the respondents, which corroborated the findings from other studies [48,49,50]. However, this prevalence is lower when compared to the 56% total consanguinity rate reported by El-Mouzan et al. The data were collected from 13 regions among the Saudi population from 2004 to 2005 [48]. The prevalence rate has significantly decreased over 16 years, going from 56% in 2004 to 2005 to 46.7% in 2019 to 2020, based on the present study’s findings. However, due to a sampling limitation by regions where most of the respondents (77.1%) in the current study are from Riyadh, the capital of Saudi Arabia, this comparison may not accurately represent the actual decline in consanguinity. 

Despite this decline, consanguineous marriage is still high in Saudi Arabia [47,48,51]; thus, strengthening information dissemination about the associated risks of consanguineous marriage must be prioritized. It was reported in the previous study that healthcare practitioners, especially multiethnic healthcare providers, had a serious knowledge gap in genetics and counseling. This is evident in their attitudes and practices regarding basic genetic counseling [51]. Therefore, more undergraduate and graduate-level medical and nursing education and training, should be required in counseling consanguineous couples. Likewise, consanguinity counseling should be incorporated into the Kingdom’s current premarital screening and genetic counseling programs, including necessary genetic literacy instruction across primary and higher education curricula.

With a better and a more focused education, which may be delivered as early as feasible, the gap in genetic knowledge and attitudes toward genetic testing among Saudis can be prevented and modified. Similarly, incorporating additional awareness programs and campaigns for the adult and senior populations may help to change people’s opinions about genetic testing. Our study and other studies prove that higher educational attainment promotes a more positive attitude toward genetic testing. On the other hand, the general awareness, and favorable attitudes of at least 50% of the population toward genetics and premarital genetic testing, revealed a bright future outlook for the population’s perception of genetics. This is supported by society’s acceptance of people with genetic abnormalities, which a little more than half of the participants in the research agreed on. 

There are certain limitations to this study that should be noted. This study exhibits selection bias because data gathering was limited to “public” locations, such as universities, hospitals, and libraries due to restrictions in going from house to house or private organizations. For instance, college students aged between 30 and 40 were projected to make up most of the samples from universities and libraries. Although different age-based samples are typically found at malls and hospitals, most of the respondents who agreed to participate in this study were primarily students and younger adults under 30. This enthusiastic response from the younger generation may be attributable to their favorable attitudes toward genetics and genetic testing and their likely comprehension of the value of the information obtained from research surveys. It is also essential to remember that almost two-thirds of Saudi Arabia’s population is under the age of 35, according to the General Authority for Statistics (GAS-TAT) Report (2020). The Kingdom’s population comprises 36.7% people aged from 15 to 34 and 30.3% people aged from 0 to 14. [52] The higher number of younger Saudis may explain the more significant proportion of responders in this age group. Another possible limitation of this study is that individuals interested in genetics volunteered more than others. Additionally, those who filled out the survey may hold a more positive attitude than those who were unwilling to participate in this study, which could lead to sample bias. Another drawback is recall bias, as several genetic ideas were taught long ago, particularly among respondents over 30. In addition, this would have been valuable data if we had included this percentage of the respondents who had formally learned genetics from universities or other institutions.

## 5. Conclusions

This study highlights public knowledge of genetics and attitudes toward genetic testing. Overall, peoples’ knowledge of genetics is nearly adequate and generally demonstrates a positive attitude towards genetic testing. Many study participants still have questions regarding genes and inheritance, even if most respondents have an adequate understanding of genetics. These questions must be addressed as soon as feasible by the curricular inclusion of basic genetic concepts. Since a higher genetic knowledge and positive attitudes toward genetic testing are significantly correlated, basic genetic concepts must be offered across curricula in higher and post-graduate education to enhance positive attitudes toward genetic testing.

Additionally, there is a need to improve public awareness of the risks and benefits of genetic testing. Genetic literacy may eventually create a positive public perception that will augment the support for genetic testing within Saudi Arabia and hopefully reduce the occurrence and burden of inherited disorders. Furthermore, while the participants know that consanguineous marriage increases the chance of having children with genetic diseases, current data suggest that consanguineous marriages in Saudi Arabia are still high, as much of the research has shown. Thus, impetus on how to disseminate genetic information, related to consanguinity and transmission of diseases should be prioritized, especially in regions where consanguine marriage rates are high. 

## Figures and Tables

**Table 1:** Sociodemographic Characteristics of Respondents (N = 1008).

Socio-Demographic Characteristics	Frequency	Percentage
**Age**		
Below 30-years old	655	65
30–60-years old	343	34
Above 60-years old	10	1
**Gender**		
Male	475	47.1
Female	533	52.9
**Current Level of Education**		
Didn’t study	9	0.9
Elementary	18	1.8
Middle school	33	3.3
High school	204	20.2
Under-graduate	641	63.6
Post-graduate	103	10.2
**Marital status**		
Single	624	61.9
Married	337	33.4
Divorced	37	3.7
Widow/widower	10	1.0
**Family Income per month**		
Enough	493	48.9
Enough and allows saving	388	38.5
Not enough	83	8.2
Not enough and leads to financial debt	44	4.4
**Residence**		
Riyadh	777	77.1
Other Regions	231	22.9
**Consanguineous Marriage**		
Born from Consanguineous Marriage	471	46.7
Born from Non-Consanguineous Marriage	537	53.3

**Table 2:** People’s General Knowledge of Genetics (N = 1008).

Statements	% of Correct Answers	% of Incorrect Answers	“I Am Not Sure Answers”
1. It is possible to see a gene with the naked eye. (False)	71.2	26.2	2.6
2. Genes control the characteristics we inherit from our parents. (True)	78.4	19.3	2.3
3. All body parts have the same genes. (True)	21.3	70.4	8.2
4. Each of us has variations in our genes that make it more likely to get certain diseases. (True)	29.2	61.4	9.4
5. Half of your genes come from your mother and a half from your father. (True)	66.5	28.7	4.8
6. Healthy parents can have a child with an inherited disease. (True)	70.4	26.4	3.1
7. The carrier of a disease gene may be completely healthy. (True)	65.7	29.2	4.7
8. If a person is a carrier of a disease gene, it means that they have the disease. (False)	60.6	35.8	3.5
9. The child of a disease gene carrier is always also a carrier of the same disease. (False)	41.4	51.8	6.8
10. All serious diseases (diseases that could be disabling or fatal at an early age), are here ditary. (False)	39.2	53.3	7.5
11. If your close relatives have diabetes or cancer, you are more likely to develop the disease. (True)	71.4	23.7	4.9
12. The onset of certain diseases not only depends on genes but also environment and lifestyle. (True)	70.3	28.4	1.2
13. Some genetic diseases can be controlled by following a healthy lifestyle. (True)	71.0	25.2	3.8
14. A person’s race and ethnicity can affect how likely they will get a disease. (True)	61.5	34.4	4.1
15. Consanguineous marriages increase the risk of having a child with a genetic disease. (True)	80.2	17.6	2.2

**Table 3:** Participants’ Attitudes Toward Genetic Testing (N = 1008).

Statements	% Agree to Strongly Agree	% Disagree to Strongly Disagree
1. I am interested in my genetic predisposition to diseases.	86.6	13.4
2. I would take a genetic profiling test to know whether I am at risk of developing diseases.	81.0	19.0
3. I would get tested only for disorders that are considered treatable or preventable.	61.4	38.5
4. I would consider having my newborn child genetically tested to learn which diseases they may develop in adulthood.	82.3	17.7
5. I am afraid that the results of a genetic test may fall into the wrong hands.	58.3	41.7
6. I am worried that due to genetic testing, disabled people will be less accepted in our society.	40.4	59.6
7. I am apprehensive that a genetic test result may result in cancelation of marriage.	42.4	57.6
8. All couples planning a pregnancy should have a possibility to have a carrier test.	88.6	11.3
9. Carrier testing will lead to higher anxiety among women who want to become pregnant.	63.2	36.8
10. Carrier testing for some diseases may lead to an inferior image of people affected with these diseases.	44.8	55.2
11. Everyone should be able to decide whether or not to undergo carrier testing.	70.0	30.0
12. It is irresponsible for couples who are willing to have children to refuse carrier testing.	76.0	24.0

**Table 4:** Association Between Genetic Knowledge and Attitude Toward Genetic Testing with Demographic Characteristics (N = 1008).

Socio-Demographic Characteristics	Knowledge in Genetics				Attitude to Genetic Testing		
	Mean	SD	*p*-Value		Mean	SD	*p*-Value
**Gender**			0.001 *				0.001 *
Male	9.371	0.123		2.677	0.015		
Female	10.026	0.102		2.776	0.014		
**Age**			0.035 *				0.166
Below 30	9.815	0.098		2.745	0.013		
30–60	9.889	0.144		2.696	0.185		
Above 60	9.900	0.983		2.725	0.115		
**Level of Education**			0.001 *				0.065
Elementary	8.333	0.498		2.694	0.084		
Middle School	8.576	0.413		2.654	0.047		
High School	9.138	0.192		2.681	0.023		
College	9.963	0.096		2.847	0.129		
Post-graduate	10.146	0.246		2.734	0.034		
Did not study	7.444	0.669		0.583	0.108		
**Marital Status**			0.016 *				0.098
Single	9.774	0.102		2.744	0.013		
Married	9.664	0.139		2.707	0.018		
Divorced	9.540	0.392		2.673	0.048		
Widow	8.600	0.618		2.592	0.110		
**Family income**			0.001 *				0.001 *
Enough & allow saving	9.525	0.115		2.712	0.015		
Enough	10.235	0.118		2.780	0.017		
Not enough	8.952	0.278		2.639	0.032		
**Residence**			0.063				0.047 *
Riyadh	9.746	0.089		2.734	0.012		
Other Regions	9.619	0.178		2.705	0.021		
**Family History:**			0.043 *				0.014 *
Born from consanguineous Marriage	9.607	0.119		2.697	0.015		
Non-consanguineous marriage	9.814	0.108		2.755	0.014		

* Significant at *p* ≤ 0.05.

**Table 5:** Multiple Regression Results between the Sociodemographic Characteristics of the Respondents and their Knowledge of Genetics and Attitudes to Genetic Testing.

Socio-Demographic Characteristics	Knowledge in Genetics				Attitudes to Genetic Testing			
	Coefficients	Std. Error	*t*-Value	*p*-Value	Coefficients	Std. Error	*t*-Value	*p*-Value
*(Constant)*	6.430	0.682	9.428	0.000	2.467	0.089	27.864	0.000
*Age*	0.003	0.010	0.318	0.751	−0.002	0.001	−1.183	0.237
*Gender*	0.778	0.163	4.787	0.000 **	0.103	0.021	4.856	0.000 **
*Current Level of* *Education*	0.526	0.105	4.997	0.000 **	0.031	0.014	2.236	0.026 **
*Marital Status*	−0.115	0.171	−0.669	0.504	−0.015	0.022	−0.684	0.494
*Family Income* *(per Month)*	−0.001	0.100	−0.010	0.992	−0.010	0.013	−0.739	0.460
*Place of Origin (Riyadh or Other Regions)*	−0.080	0.192	−0.417	0.677	−0.011	0.025	−0.438	0.662
*Born from Consanguineous or Non-Consanguineous Marriage*	0.178	0.158	1.128	0.259	0.054	0.020	2.637	0.009 **
	**Knowledge in Genetics**				**Attitudes to Genetic Testing**			
	**Regression**	**Residual**		**Total**	**Regression**	**Residual**		**Total**
*df*	7	1000		1007	7	1000		1007
*Sum of Squares*	286.550	6223.869		6510.419	4.761	104.926		109.687
*R^2^*		0.044		0.043		0.044		0.043
*R^2^ (adj)*		0.037		0.037		0.037		0.037
*F*		6.577		6.483		6.577		6.483
*Sig*		0.000 **		0.000 **		0.000 **		0.000 **

** Significant at *p* ≤ 0.05.

## Data Availability

The data used in this study are available and will be provided by the corresponding author at a reasonable request.
